# Pharmacokinetic interaction between rhynchopylline and pellodendrine via CYP450 enzymes and *P-gp*

**DOI:** 10.1080/13880209.2021.1999988

**Published:** 2021-11-10

**Authors:** Qingzhen Meng, Yongheng Cheng, Cui Zhou

**Affiliations:** a Department of Intravenous Drug Allocation, Weifang Maternal and Child Health Hospital, Weifang, China; b Outpatient Pharmacy, Weifang Maternal and Child Health Hospital, Weifang, China

**Keywords:** *Uncaria rhynchophylla* (Miq.) Miq. ex Havil, *Phellodendron amurense* Rupr, herb–herb interaction

## Abstract

**Context:**

Rhynchopylline and pellodendrine are major extractions of commonly used Chinese medicine in gynaecology. The interaction between these two compounds could affect treatment efficiency and even result in toxicity during their co-administration in gynaecological prescription.

**Objective:**

The pharmacokinetic interaction between rhynchopylline and pellodendrine and the potential mechanism were investigated in this study.

**Materials and methods:**

Sprague-Dawley rats were randomly divided into four groups to investigate the pharmacokinetic interaction between rhynchopylline (30 mg/kg) and pellodendrine (20 mg/kg) with single dose of these two drugs as the control. The transport of rhynchopylline was evaluated in the Caco-2 cell model. Additionally, the metabolic stability and the activity of corresponding CYP450 enzymes were assessed in rat liver microsomes.

**Results:**

The pharmacokinetic profile of rhynchopylline was dramatically affected by pellodendrine with the increased area under the pharmacokinetic curve (3080.14 ± 454.54 vs. 1728.08 ± 220.598 μg/L*h), *C*_max_ (395.1 ± 18.58 vs. 249.1 ± 16.20 μg/L), prolonged *t*_1/2_ (9.74 ± 2.94 vs. 4.81 ± 0.42 h) and the reduced clearance rate (from 11.39 ± 1.37 to 5.67 ± 1.42 L/h/kg). No significant changes were observed in the pharmacokinetics of pellodendrine. The transport of rhynchopylline was significantly inhibited by pellodendrine with a decreasing efflux ratio (1.43 vs. 1.79). Pellodendrine significantly inhibited the activity of CYP1A2 and CYP2C9 with IC_50_ values of 22.99 and 16.23 μM, which are critical enzymes responsible for the metabolism of rhynchopylline.

**Discussion and conclusions:**

The adverse interaction between rhynchopylline and pellodendrine draws attention to the co-administration of these two herbs and provides a reference for further investigations with a broader study population.

## Introduction

In traditional Chinese medicine, herbs that possessed similar indications usually exist in the same prescription, so that they may complement each other in the treatment of multiple disease symptoms and improve the therapeutic efficiency. *Uncaria rhynchophylla* (Miq.) Miq. ex Havil. (Rubiaceae) and *Phellodendron amurense* Rupr. (Rutaceae) are two main herbs in the gynecological prescription for such problems as pregnancy eclampsia and cervicitis, and have been demonstrated to have the pharmacological activities of antibacterial, anti-inflammation, and detoxification. The activity of major ingredients in herbs is critical for displaying their pharmacological effects.

Rhynchopylline is a major extraction of *Uncaria rhynchophylla* branch and has been applied as a component of various medications, such as *Choto-san* (Shellard and Lala 1978). Numerous studies have been conducted to disclose the pharmacological actions of rhynchopylline. For example, Hongyan et al. (2019) found rhynchopylline could alleviate the neurotoxicity induced by Tourette syndrome by regulating the TLR/NLRP3/NF-κB pathway. Rhynchopylline was also revealed to possess a neuroprotective effect in Alzheimer’s disease (Xu et al. 2020).

Phellodendrine is an important characteristic ingredient of *Phellodendron amurense* and contains two phenolic groups. In a previous study, pellodendrine was suggested to act as an immunosuppressor to relieve cell immune response (Mori et al. 1995). The antitumor activity of pellodendrine has also been reported in pancreatic cancer, and it also has an anti-inflammatory effect to treat ulcerative colitis and nephritic (Hattori et al. 1992; Thu et al. 2019; Su et al. 2021).

Interaction between active compounds in co-administrated herbs is a vital factor that affects the medicinal effect of herbs. The pharmacokinetic study is one of the most important means to assess clinical efficiency, guide rational herb co-administration, and improve clinical medication strategy. Several pharmacokinetic studies revealed the interaction between different drugs or herbs. For example, the co-administration of bazedoxifene and ibuprofen results in the increased bazedoxifene plasma concentration indicating the drug interaction between bazedoxifene and ibuprofen (McKeand et al. 2018). Curcumin inhibited the metabolism of amlodipine, which prolonged the system exposure and plasma concentration of amlodipine by inhibiting the activity of CYP3A4 (Jiang et al. 2020). The activity of cytochrome P450 enzymes is a major inducing factor during the combination of different drugs. Phellodendrine has been demonstrated to inhibit the activity of CYP1A2, 3A4, and 2C9, which implies its potential to interact with the drugs metabolized by these CYPs (Li et al. 2020). As several CYPs are involved in the metabolism of rhynchopylline, the co-administration of rhynchopylline and pellodendrine might induce adverse interaction and even result in herb toxicity (Wang et al. 2010). Therefore, it is important to investigate the co-administration of rhynchopylline and pellodendring. This may provide guidance for the clinical application of their origin herbs.

## Materials and methods

### Animals and grouping

This study was approved by the Ethics Committee of Weifang Maternal and Child Health Hospital (2019034). Male Sprague-Dawley rats (230–250 g, Bikei Animal Company, China) were applied in this study. All animals were housed in controlled conditions (22 ± 2 °C with a relative humidity of 50 ± 10%) and had free access to a standard diet. The conformation of the animals was conducted for 5 days. Before the experiments, rats were fasted but had free access to water for 12 h.

The rats were randomly divided into four groups with six rats of each as follows:

Group A: orally administration of 30 mg/kg rhynchopylline (purity > 98%, Chinese Biopharmaceutical Institute, China)
Group B: pre-treatment of 20 mg/kg pellodendrine (purity > 98%, Chinese Biopharmaceutical Institute, China) for 7 days followed by the administration of 30 mg/kg rhynchopylline
Group C: orally administration of 20 mg/kg pellodendrine
Group D: pre-treatment of 30 mg/kg rhynchopylline followed by the administration of 20 mg/kg pellodendrine

The dosage used in the experiments were selected according to previous studies (Yang et al. 2017; Chen et al. 2018).

### Pharmacokinetic study and sample preparation

The plasma samples were collected after a period (0, 0.25, 0.5, 1, 2, 3, 4, 6, 8, 12, 24, 36, and 48 h) of the administration of rhynchopylline or pellodendrine. The plasma samples were collected in a heparinized tube with the ocular choroidal vein. The collected samples were mixed with methanol and internal standard methanol solution (2 ng/mL) and vortexed for 60 s. After centrifugation for 10 min at 12000 rpm, the supernatant was reserved and stored at −80 °C for the following analyses.

### Instrumentation and conditions

An Agilent 1290 series liquid chromatography system and the Agilent 6460 triple-quadruple mass spectrometer were used for the detection of rhynchopylline and pellodendrine. The obtained data were analyzed with the Agilent Quantitative analysis software.

The samples were separated on the Waters Xbridge C18 column. The mobile phase was 0.1% formic acid and acetonitrile (65:35, v/v, Fisher Scientific, USA), and the column temperature was 25 °C. The flowing rate was 0.4 mL/min with the injection volume of 5 μL. MRM mode of *m/z* 385.2→160.1 for rhynchopylline and *m/z* 343.2→193.2 for phellodendrine.

### Evaluation of rhynchopylline transport in Caco-2 cell transwell model

Caco-2 cells were obtained from ATCC and cultured in DMEM high glucose medium (Thermo Scientific, USA) with 10% foetal bovine serum (GIBCO BRL, USA), 1% non-essential amino acid (Thermo Scientific, USA), and 1% penicillin and streptomycin (Amresco, USA) at 37 °C with 5% CO_2_.

The cultured cells were seeded onto the transwell polycarbonate insert filters with a cell density of 1 × 10^5^ cell/cm^2^ and incubated for 21 d. The culture medium was replaced every two days in the first seven days, and then daily. When the paracellular flux of Lucifer yellow was less than 1%/h, the Caco-2 monolayers were available for the transport study.

The cell monolayers were preincubated for 20 min at 37 °C and then incubated with rhynchopylline (both the apical and basolateral sides). Phellodendrine or the positive inhibitors of *P-gp* was added to both sides of the cell monolayers and preincubated for 30 min at 37 °C. The mixture was collected at the indicated time points, and the same volume of fresh HBSS buffer was added to keep the total volume.

The permeability of rhynchopylline was evaluated after 2 h of the incubation and the corresponding parameters were calculated with the following equation:

$$P_{\mathrm{app}}=(\Delta Q/\Delta t)\times [1/(A\times C_{0})]$$

where P_app_ is the apparent permeability coefficient (cm/s), Δ*Q*/Δ*t* (μmol/s) is the rate at which the compound appears in the receiver chamber, *C*_0_ (μmol/L) is the initial concentration of the compound in the donor chamber and *A* (cm^2^) represents the surface area of the cell monolayer. Data were collected from three separate experiments, and each was performed in triplicate.

### In vitro *metabolic experiment in rat liver microsomes*

The rat liver microsome, rhynchopylline, and PBS buffer were mixed in the centrifuge tubes on ice. The reaction was initiated by an NADPH-generating system after preincubation at 37 °C for 5 min. To investigate the effect of phellodendrine, a preincubation with phellodendrine was performed for 30 min at 37 °C. After 0, 5, 15, 30, 45, and 60 min of the reaction, acetonitrile was added to terminate the reaction and the mixture was collected to analyze the concentration of rhynchopylline by LC-MS/MS.

The corresponding parameters were calculated by the following equations:

$$t_{1/2}=0.693/k;$$

V (μL/mg)=volume of incubation (μL)/protein in the incubation (mg);

$$\text{Intrinsic clearance }(\mathrm{Clint})\;(\mu L/\min/\text{mg protein})\;=\;V\times 0.693/t_{1/2}.$$

### In vitro *evaluation of CYP450 activity in rat liver microsomes*

The activity of CYP1A2 and 2C9 was evaluated with corresponding typical substrates and various concentrations of phellodendrine (0, 2, 5, 10, 25, 50, and 100 μM) in rat liver microsomes according to a previous study (Li et al. 2020). The activity of CYP1A2 and 2C9 was assessed by the remaining concentration of substrates analysed by HPLC.

### Statistical analysis

The pharmacokinetic parameters were obtained with the help of DAS 3.0 pharmacokinetic software (Chinese Pharmacological Association, China). The data were represented as mean value ± SD. and analyzed with one-way ANOVA followed by Turkey *post hoc* test. *p* < 0.05 indicates the statistically significant difference.

## Results

### Pharmacokinetic interaction between pellodendrine and rhynchopylline

**Figure 1.**
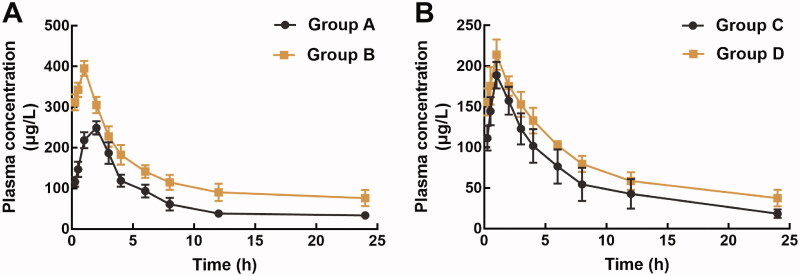
The pharmacokinetic profiles of rhynchopylline (A) and pellodendrine (B) in different groups. Group A: orally administration of 30 mg/kg rhynchopylline; Group B: pre-treatment of 20 mg/kg pellodendrine for 7 days followed by 30 mg/kg rhynchopylline; Group C: orally administration of 20 mg/kg pellodendrine; Group D: pre-treatment of 30 mg/kg rhynchopylline followed by 20 mg/kg pellodendrine.

The pharmacokinetic profile of pellodendrine and rhynchopylline was investigated respectively to evaluate their potential interaction. The pharmacokinetic profile of rhynchopylline was dramatically affected by pellodendrine (Figure 1(A)). Specifically, pellodendrine significantly increased the area under the curve (*AUC*, 3080.14 ± 454.54 vs. 1728.08 ± 220.598 μg/L/h) and the maximum concentration (*C*_max_, 395.1 ± 18.58 vs. 249.1 ± 16.20 μg/L) of rhynchopylline (Table 1). Meanwhile, the presence of pellodendrine prolonged the half-life (*t*_1/2_, 9.74 ± 2.94 vs. 4.81 ± 0.42 h) and significantly reduced the clearance rate of pellodendrine (*Cl/F*, from 11.39 ± 1.37 to 5.67 ± 1.42 L/h/kg).

**Table 1.** Corresponding pharmacokinetic parameters of rhynchopylline (Group A and B) and pellodendrine (Group C and D) in different groups.

	Unit	Group A	Group B	Group C	Group D
AUC _(0-_*_t_*_)_	μg/L*h	1728.08 ± 220.598	3080.14 ± 454.54*	1416.58 ± 362.93	1914.68 ± 259.94
*t*_1/2_	h	4.81 ± 0.42	9.74 ± 2.94*	10.25 ± 2.88	11.02 ± 2.56
*T*_max_	h	1.83 ± 0.22	0.87 ± 0.24*	1.03 ± 0.26	0.94 ± 0.13
Cl/F	L/h/kg	11.39 ± 1.37	5.67 ± 1.42*	12.34 ± 2.76	8.44 ± 1.59
*C*_max_	μg/L	249.1 ± 16.20	395.1 ± 18.58*	188.9 ± 16.22	214.07 ± 18.67

*p < 0.05.

No significant change was observed in the pharmacokinetic profile of pellodendrine during the co-administration of pellodendrine and rhynchopylline (Figure 1(B)). A weak increase was found in the *AUC* and *C*_max_ of pellodendrine, but the difference was not significant. Similar trends were also observed in the *t*_1/2_ of pellodendrine, but a slight decrease was found in the Cl/F of pellodendrine, and all differences were not significant (Table 1).

**Figure 2.**
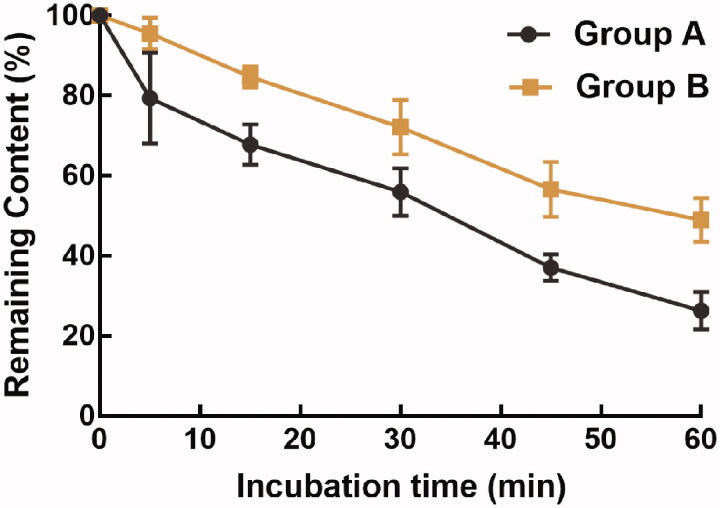
The metabolic stability of rhynchopylline in the presence or absence of pellodendrine.

Consistently, the half-life of rhynchopylline in rat liver microsomes was 35.67 min with the intrinsic clearance rate of 38.86 mL/min/kg protein, and pellodendrine prolonged the half-life to 58.01 min and decreased the intrinsic clearance to 23.89 mL/min/kg protein (Figure 2).

### The transport of pellodendrine and rhynchopylline

**Figure 3.**
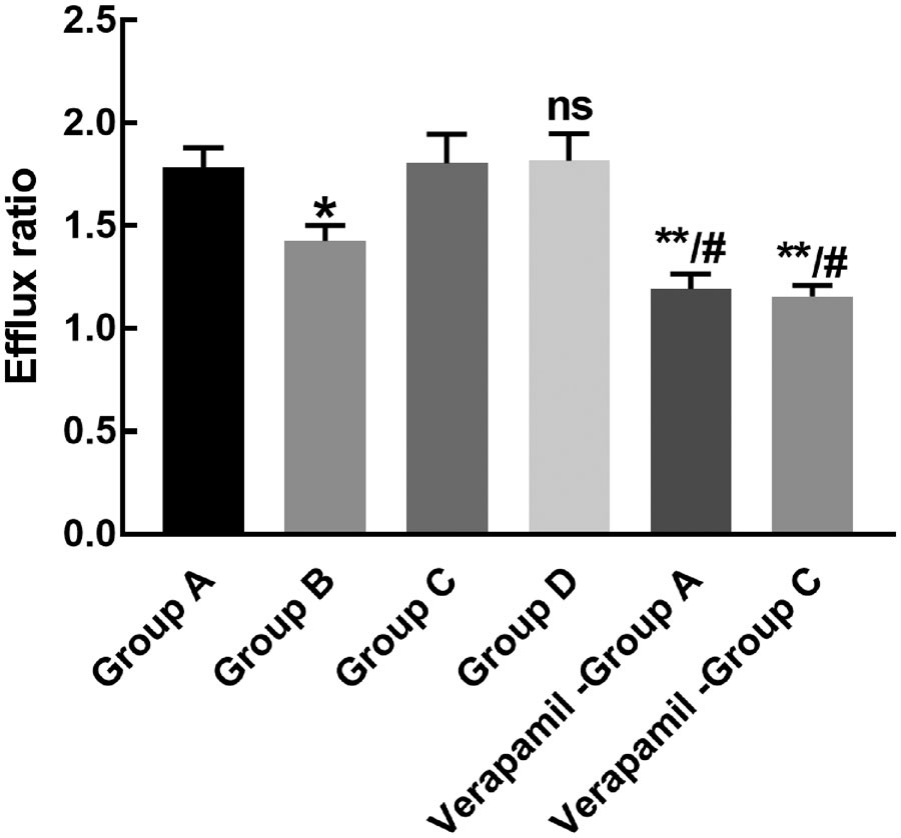
The transport of rhynchopylline and pellodendrine in the Caco-2 cell model in different groups. *p < 0.01, **p < 0.001 relative to Group A; ^#^*p* < 0.05 relative to Group A or Group C.

The efflux ratio of both rhynchopylline and pellodendrine was significantly decreased in the presence of verapamil, indicating the potential involvement of *P-gp* in their transport. The efflux ratio of rhynchopylline was dramatically inhibited by the co-administration of pellodendrine (*p* < 0.05, Figure 2). The efflux ratio of pellodendrine was not affected by the co-administration of rhynchopylline (*p* > 0.05, Figure 3).

### Pellodendrine inhibited the activity of CYP1A2 and CYP2C9 in a dose-dependent manner

**Figure 4.**
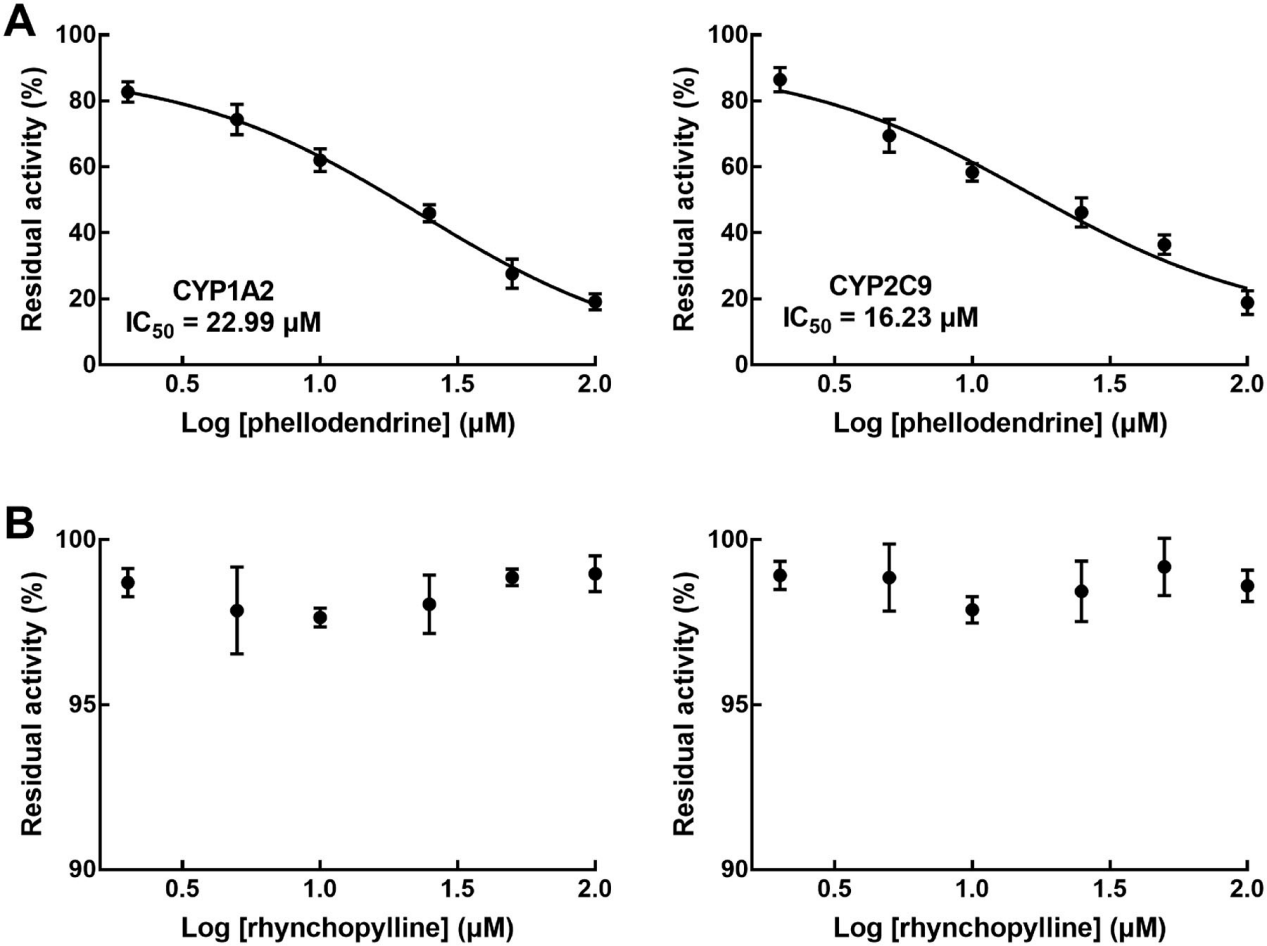
Effect of pellodendrine and rhynchopylline on the activity of CYP1A2 and CYP2C9. (A) pellodendrine inhibited the activity of CYP1A2 and CYP2C9 in a dose-dependent manner with the IC_50_ values of 22.99 and 16.23 μM. (B) Rhynchopylline did not affect the activity of CYPs.

In rat liver microsomes, phellodendrine showed a significant inhibitory effect on the activity of CYP1A2 and CYP2C9 with the IC_50_ values of 22.99 and 16.23 μM, respectively (Figure 4(A)). The activity of CYPs was not influenced in the presence of different concentrations of rhynchopylline (Figure 4B).

## Discussion

Herb-herb interaction commonly occurs during the co-administration of different herbs, which would mimic, magnify, or oppose the effect of drugs (Chen et al. 2020). Therefore, herb-herb investigations should be given special attention. Pellodendrine and rhynchopylline are two major ingredients of commonly used herbs in the prescription of gynecological diseases. The interaction between these two compounds was estimated in the present study to guide the clinical combination of their original herbs.

During the co-administration of pellodendrine and rhynchopylline, the pharmacokinetic profile of rhynchopylline was significantly changed in the presence of pellodendrine with increased *AUC*, prolonged *t_1/2_*, and decreased clearance rate. The pharmacokinetics of pellodendrine showed no significant difference between single-dose and co-administration with rhynchopylline. Consistent with the *in vivo* results, pellodendrine enhanced the metabolic stability of rhynchopylline with increased half-life and decreased intrinsic clearance rate. In previous investigations, the activity of corresponding metabolic enzymes, such as CYP450s, was considered as one of the main factors that lead to adverse herb-herb interactions. For example, Panax ginseng was reported to exert interaction with various drugs or herbs, such as warfarin and antidepressants, due to its inducing effect on the activity of CYP3A and P-gp (Fugh-Berman 2000; Ramanathan and Penzak 2017). CYP1A2 and CYP2C9 are two major enzymes participating in the metabolism of rhynchopylline (Wang et al. 2010). To investigate the mechanism underlying the interaction between rhynchopylline and pellodendrine, the effect of pellodendrine and rhynchopylline on the activity of CYP1A2 and CYP2C9 was estimated in rat liver microsomes. The significant inhibitory effect of pellodendrine on the activity of CYP1A2 and CYP2C9, while rhynchopylline did not affect the activity of CYPs, which was consistent with a previous study (Li et al. 2020). Therefore, the inhibition of CYP1A2 and CYP2C9 was speculated as the potential cause for the increased system exposure of rhynchopylline by pellodendrine. The pharmacokinetic parameters of pellodendrine showed weak changes, but the difference was insignificant. This might result from the potential competition between pellodendrine and rhynchopylline for the metabolic enzymes, which needs further validation.

*P-gp* is a key protein that is responsible for the transport of various drugs and mediates the interaction between different drugs (Elmeliegy et al. 2020). In the Caco-2 cell model, the *P_appBA_* value of rhynchopylline was much larger than *P_appAB_* indicating the involvement of P-gp in the transport of rhynchopylline. Meanwhile, the transport of rhynchopylline was also observed to be suppressed by pellodendrine, which might be the result of the inhibition of *P-gp*. No similar effects were found in the transport of pellodendrine.

## Conclusions

Taken together, pellodendrine significantly increased the plasma concentration of rhynchopylline and prolonged its systemic exposure. The pharmacokinetics of pellodendrine was not affected by the co-administration with rhynchopylline. The demonstrated inhibitory effects of pellodendrine on CYP1A2, CYP2C9, and *P-gp* were the direct mechanism underlying the interaction between these two compounds.

## Data Availability

Corresponding authors may provide relevant data if appropriate.
